# Live-cell imaging with *Aspergillus fumigatus*-specific fluorescent siderophore conjugates

**DOI:** 10.1038/s41598-020-72452-2

**Published:** 2020-09-23

**Authors:** Joachim Pfister, Alexander Lichius, Dominik Summer, Hubertus Haas, Thines Kanagasundaram, Klaus Kopka, Clemens Decristoforo

**Affiliations:** 1 Department of Nuclear Medicine, Medical University Innsbruck, Innsbruck, Austria; 2 Department of Microbiology, University Innsbruck, Innsbruck, Austria; 3 Division of Molecular Biology, Medical University Innsbruck, Innsbruck, Austria; 4 Division of Radiopharmaceutical Chemistry, German Cancer Research Center, (DKFZ), Im Neuenheimer Feld 280, 69120 Heidelberg, Germany; 5 Institute of Radiopharmaceutical Cancer Research, Helmholtz-Zentrum Dresden-Rossendorf, Dresden, Germany; 6 Faculty of Chemistry and Food Chemistry, Technische Universität Dresden, Dresden, Germany

**Keywords:** Imaging, Microscopy, Biomarkers, Molecular medicine

## Abstract

Live-cell imaging allows the in vivo analysis of subcellular localisation dynamics of physiological processes with high spatial–temporal resolution. However, only few fluorescent dyes have been custom-designed to facilitate species-specific live-cell imaging approaches in filamentous fungi to date. Therefore, we developed fluorescent dye conjugates based on the sophisticated iron acquisition system of *Aspergillus fumigatus* by chemical modification of the siderophore triacetylfusarinine C (TAFC). Various fluorophores (FITC, NBD, Ocean Blue, BODIPY 630/650, SiR, TAMRA and Cy5) were conjugated to diacetylfusarinine C (DAFC). Gallium-68 labelling enabled in vitro and in vivo characterisations. LogD, uptake assays and growth assays were performed and complemented by live-cell imaging in different *Aspergillus* species. Siderophore conjugates were specifically recognised by the TAFC transporter MirB and utilized as an iron source in growth assays. Fluorescence microscopy revealed uptake dynamics and differential subcellular accumulation patterns of all compounds inside fungal hyphae.[Fe]DAFC-NBD and -Ocean Blue accumulated in vacuoles, whereas [Fe]DAFC-BODIPY, -SiR and -Cy5 localised to mitochondria. [Fe]DAFC -FITC showed a uniform cytoplasmic distribution, whereas [Fe]DAFC-TAMRA was not internalised at all. Co-staining experiments with commercially available fluorescent dyes confirmed these findings. Overall, we developed a new class of fluorescent dyes that vary in intracellular fungal targeting , thereby providing novel tools for live-cell imaging applications for *Aspergillus fumigatus*.

## Introduction

Live-cell imaging is the key technology to analyse physiological processes inside filamentous fungi, including the uptake and distribution dynamics of fluorescent dyes. The ability to visualize subcellular structures and follow developmental processes in vivo presents a major advantage over fixed samples. Confocal laser scanning microscopy is the most widely available and thus routinely used technology to image and quantify physiological activities with high spatial and high temporal resolution^1^. The combination of genetically encoded fluorescent fusion proteins with organelle-specific fluorescent dyes grants experimental access to virtually any cellular process. Also rapid advances in computer hard- and software now allow the processing of vast amounts of data, particularly those produced during multi-colour 3D-time-lapse recordings^2,3^.

Although a considerable number of organelle-specific fluorescent probes are available for the visualisation of different cellular compartments in filamentous fungi^4,5^, none are species-specific and therefore they are not applicable for identification or targeted treatment in situ.

To overcome this limitation, we exploited the iron acquiring siderophore system of filamentous fungi. Iron is essential for most prokaryotes and all eukaryotes to grow and reproduce. Therefore, iron acquisition has top priority for any organism but is limited by its bioavailability. Atmospheric oxygen rapidly oxidizes the water-soluble ferrous form (Fe(II)) into ferric hydroxide (Fe(III)). Microorganisms have developed different strategies to overcome this problem. *Aspergillus fumigatus* (*A. fumigatus*), an airborne human pathogen that causes life-threatening invasive pulmonary aspergillosis (IPA) with a mortality rate of up to 90%^6,7^, has two high-affinity iron acquisition systems: reductive iron assimilation and siderophore-assisted iron uptake^8^. Siderophores are low molecular organic molecules that bind ferric iron and fulfill either an iron acquisition or internal storage function from the fungal environment^9^. *A. fumigatus* produces the hydroxamate-type siderophores desferri-fusarinine C (FsC) and desferri-N,N’,N’’-triacetylfusarinine C (TAFC) which are secreted in response to iron starvation, as well as desferri-ferricrocin (FC) and desferri-hydroxyferricrocin (HFC) which are used for internal iron handling in hyphae and conidia, respectively^9,10^. After secretion, TAFC binds ferric iron with particularly high binding affinity (pM = 31.8, TAFC^11^) and is subsequently reabsorbed via the specific energy-dependent major facilitator transporter MirB^12^*.* Uptake of [Fe]TAFC by MirB is characteristic for *A. fumigatus* as it is not recognized by most other fungi or bacteria^13,14^. Therefore, modification of TAFC is a highly promising approach for the generation of novel and accurate diagnostic biomarkers for the detection of *A. fumigatus* infections.

In a recent study, we developed strategies to synthesize different TAFC derivatives by subsequently substituting the free amines of the FsC molecule^15,16^. Uptake assays demonstrated that various modifications are possible without losing recognition of TAFC by MirB^17^. During the development of novel hybrid imaging applications for fungal infection diagnostics, combining positron emission tomography (PET) with optical imaging by introduction of a near-infrared (NIR) dye^18^, we successfully visualized *A. fumigatus* infections. However, we also observed loss of uptake depending on the fluorescent label used.

**Figure 1:**
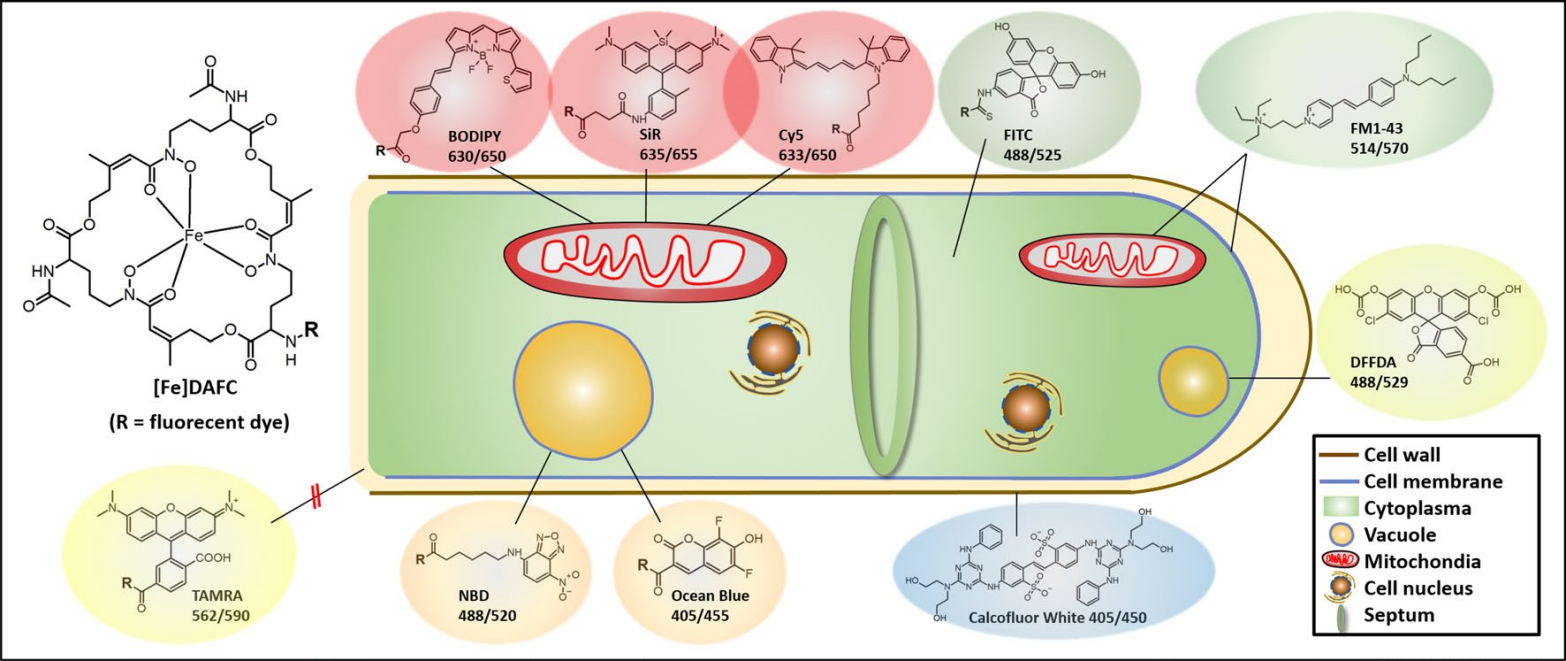
Schematic picture of an *A. fumigatus* hypha with chemical structures and identified subcellular localisations of different fluorescent [Fe]DAFC conjugates. Localisation to the cell wall, plasma membrane, mitochondria and vacuoles was confirmed by co-staining with Calcofluor White, FM 1–43 and DFFDA, respectively. Numbers indicate excitation and emission peak wavelengths of the respective fluorophores.

To investigate this effect, we broadened our scope to include additional fluorescent dyes that cover a wider spectrum of excitation wavelengths (Fig. 1), and characterized their individual uptake behaviour. The aim was to identify optimized compounds that are selectively recognized by *A. fumigatus* for applications including live-cell imaging microscopy.

Starting from the diacetylated form (diacetylfusarinine C, DAFC)^18^ we coupled a series of different fluorescent dyes and characterized their recognition by MirB.

## Methods

### Chemicals

All chemicals were purchased from commercial sources as reagent grade and used without further purification unless stated otherwise. Fluorescent dyes were obtained in their carboxylic acid form, as *N*-hydroxysuccinimid-esters or isothiocyanates for coupling and were used without further purification: Fluorescein isothiocyanate (FITC) (Sigma Aldrich, Vienna, Austria)) 6-(7-Nitrobenzofurazan-4-ylamino)hexanoic acid (NBD) (Sigma Aldrich, Vienna, Austria), 5(6)-carboxy-2′,7′-dichlor-fluorescein-D (DFFDA) (Sigma Aldrich, Vienna, Austria), Calcofluor White stain (CFW) (Sigma Aldrich, Vienna, Austria), 4,4-difluoro-4-bora-3a,4a-diaza-s-indacene (BODIPY 630/650), (Lumiprobe GmbH, Hannover, Germany), Cyanine 5-carboxylic acid (Cy5) (Lumiprobe GmbH, Hannover, Germany), 3-Carboxy-6,8-difluoro-7-hydroxycoumaryl succinimidyl ester (Ocean Blue); 5-Carboxytetramethylrhodamine-NHS (TAMRA) (Bio-Techne Ltd, Abingdon, UK), 10-(5-amino-2-methylphenyl) silicon-rhodamine (SiR) (prepared in house^19^), (*N*-(3-Triethylammoniumpropyl)-4-(4-(Dibutylamino)Styryl)pyridinium dibromide) (FM1-43) (ThermoFischer Scientific, Watham, MA, USA).

### Synthesis of the fluorescent conjugates and radiolabelling

[Fe]FsC was used as a starting material to produce [Fe]DAFC as previously described^16,20^. Fluorescent dyes were coupled depending on their different functional moiety. Carboxylic acid derivatives were activated with O‐(7‐azabenzotriazol‐1‐ yl)‐1,1,3,3‐tetramethyluronium‐hexafluorophosphate (HATU) for conjugation with the free amine of [Fe]DAFC in dimethylformamide (DMF), or already pre-activated dyes were used (isothiocyanate, NHS-ester). After reaction at room temperature and under light exclusion, the product was purified by preparative RP-HPLC to give a coloured solid powder after freeze drying. Identity was confirmed by MALDI-TOF MS (Bruker Daltonics, Bremen, Germany).

To obtain the iron free siderophores, the product was treated with a 1,000-fold excess of ethylenediaminetetraacetic acid (EDTA) at pH 4 and purified by preparative RP-HPLC, as described earlier^18^. ^68^Ga-compounds were prepared as previously described^21^ using gallium-68 from a commercial ^68^Ge/^68^Ga-generator (IGG100, Eckert & Ziegler Isotope products, Berlin, Germany) and incubating with iron free siderophore in acetate buffer at pH 4.5. The radiolabelled conjugates were used without further purification for LogD determination and uptake/competition assays, respectively. For all other experiments [Fe]-complexes were used.

More detailed chemical information and analytical conditions are provided as supplementary material.

### In vitro characterization

#### Distribution coefficient (LogD)

A 50 µL aliquot of ^68^Ga-labelled fluorophore conjugate (~ 5 µM) was added to 450 µL PBS + 500 µL n-octanol and mixed for 20 min at 1,400 rpm in a standard vortexer (MS 3, IKA, Staufen, Germany). Subsequently, the mixture was centrifuged for 2 min at 4,500 rpm (Eppendorf Centrifuge 5,424, Hamburg, Germany) and aliquots of 200 µL of each phase were measured in the gamma counter (2,480 automatic Gamma counter Wizard2 3′’, Perkin Elmer, Waltham, MA, USA). LogD was calculated as the ratio of octanol/water phase using Excel. (n = 3, six technical replicates).

#### Uptake and competition assay

Uptake assays were performed as previously described^17,18^. Briefly, 180 μL of *A. fumigatus* culture in iron‐depleted and iron‐replete media, respectively, were added in 96‐well MultiScreen Filter Plates HTS (1 μm glass fiber filter, Merck Millipore, Darmstadt, Germany) and pre‐incubated for 15 min with either PBS or [Fe]TAFC (blocking solution) at 37 °C. Subsequently, radiolabelled compound (final concentration approximately 90 nM) was added before incubation continued for 45 min at 37 °C. Hereafter hyphae were washed twice with icecold TRIS buffer (15 mM Tris(hydroxymethyl)-aminomethane) and dry filters were measured in the gamma counter. Competition assays were performed in the same way except that fungal cultures were pre‐incubated with iron‐labelled fluorophore conjugates for 15 min and the uptake value of [^68^Ga]Ga‐TAFC into hyphae was determined in order to demonstrate specific interaction with the MirB transporter.

#### Growth promotion assay

Growth promotion assays were performed as previously described^17,22^ using a mutant strain (ΔsidA/ΔftrA) of *A. fumigatus* that lacks sidA and ftrA which have siderophore production and reductive iron assimilation functions. Spores were point inoculated (104 conidia) in 24‐well plates, containing 0.5 mL of Aspergillus minimal medium agar and an increasing concentration of iron containing siderophore ranging from 0.1–50 μM. Plates were incubated for 48 h at 37 °C in a humidity chamber and visually assessed^18^. Without siderophore supplementation, no growth of this mutant strain was observed.

#### Live-cell imaging

Fluorescence microscopy was performed on a Leica TCS SP5 II inverted confocal laser scanning microscope equipped with eight excitation laser lines between 405 and 633 nm, a four-channel filter-free AOBS (Acousto‐Optical Beam Splitter) and three photo-multiplier tubes and one Leica HyD detector.

Liquid cultures of fungal germling were prepared in μ‐Slide 8 Well chambered coverslips (cat.no. 80821, ibidi GmbH, Martinsried, Germany). Each well was inoculated with 5 × 10^3^ Spores in 200 μL minimal medium and incubated at 37 °C in a humidified chamber. *A. fumigatus* (ATCC 46,645) was cultivated for 14 h and *A. terreus* (ATCC 3,633) for 48 h to obtain well developed germlings and young hyphae without extensive cell fusion. For microscopy, fluorescent dyes were used at a final concentration of 10 μM and incubated for 5–20 min. For co-staining experiments, FM1-43 (10 µM), CFW (10 µM) or DFFDA (10 µM) were added simultaneously with the siderophore conjugate. Blocking experiments with NaN_3_ (final concentration of 1 mM) and [Fe]TAFC (final concentration of 1 mM) were performed by pre-incubation of the blocking substance for 15 min before adding the fluorophore conjugate. Excitation laser intensity during image acquisition was kept to a minimum to reduce photobleaching and phototoxic effects to the cells while still achieving good signal‐to‐noise ratios. The precise image acquisition settings are shown for each conjugate in Table S1. Images were recorded with a maximum resolution of 1024 × 1024 pixels and saved as PNG. Z‐stack acquisition is indicated in the image description where applicable. Apart from brightness and contrast adjustments and cropping using the ImageJ 1.52a open source software platform (Wayne Rasband, NIH, Bethesda, MD, USA), images were not subjected to further manipulation^18^.

## Results

### Synthesis of fluorescent conjugates and radiolabelling

All fluorescent conjugates were synthesized with an excellent chemical purity (> 95%, monitored by analytical RP-HPLC, UV absorption at *λ* = 220 nm) and yields ranging from 20–40%. Corresponding mass analysis was in good agreement with the calculated values. Radiolabelling was achieved in 10 min at room temperature, with almost quantitative radiochemical yields (> 95%) (representative HPLC radiochromatograms are shown in Figure S1).

### In vitro characterization

#### LogD

Distribution coefficient experiments revealed a high variation in lipophilicity, from 0.33 for [^68^Ga]GaDAFC-BODIPY to -1.86 for [^68^Ga]GaDAFC-Ocean Blue (Table 1).

**Table 1:** Distribution coefficient (octanol/water) of siderophore compounds radiolabelled with gallium-68. Experiments were repeated three times with six technical replicates. (*) Data from^18^.

	DAFC-FITC	DAFC-NBD	DAFC-ocean blue	DAFC-BODIPY	DAFC-SiR	DAFC-TAMRA	DAFC-Cy5
Log D (pH 7.4)	− 1.75 ± 0.21	− 0.15 ± 0.01	− 1.86 ± 0.03	0.33 ± 0.06	0.24 ± 0.001	− 1.13 ± 0.02	1.03 ± 0.105*

Data are presented as mean ± SD.

### In vivo characterization

#### In vitro uptake of ^68^Ga-siderophores

**Figure 2:**
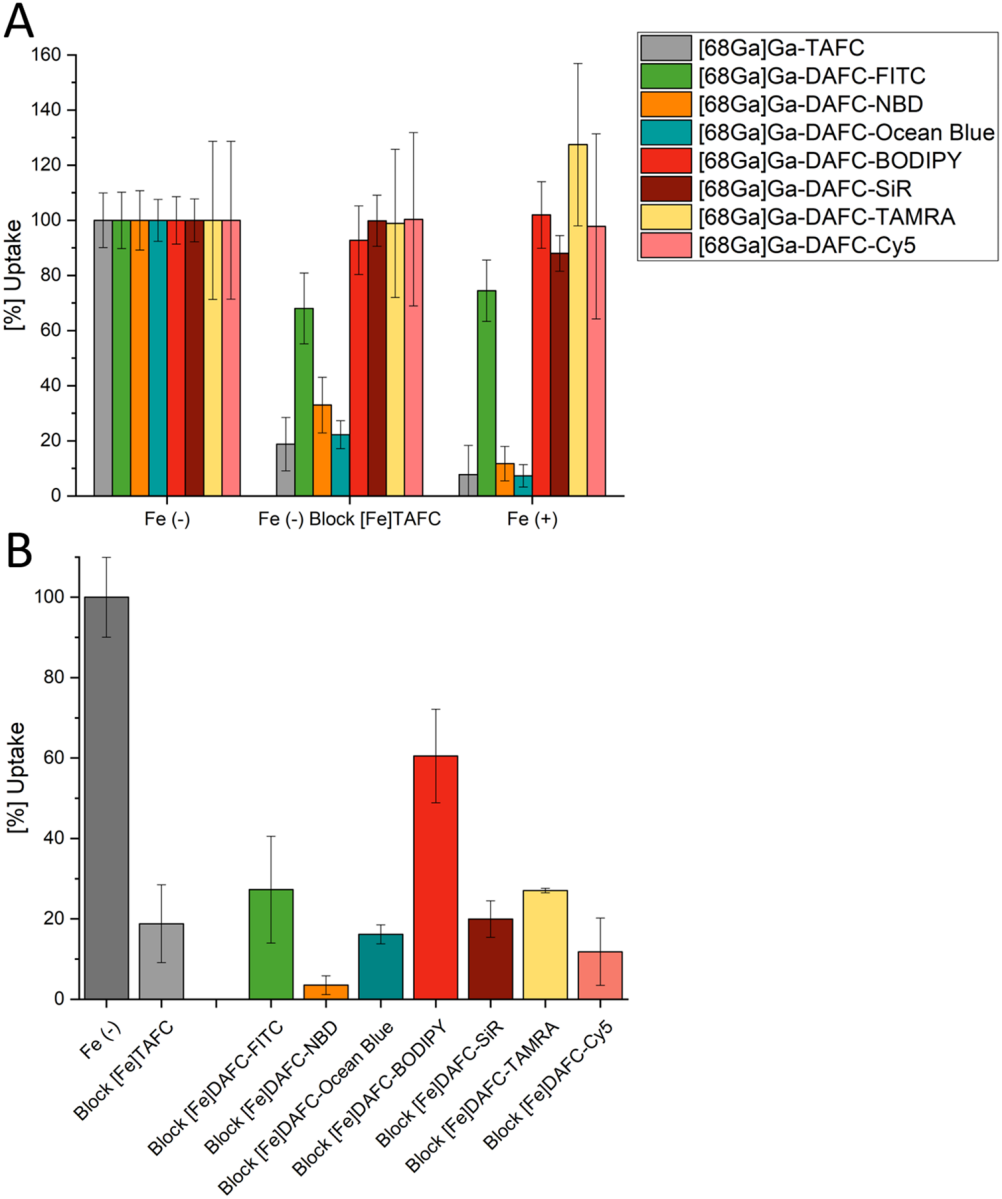
(**A**) Uptake of different radiolabelled, fluorescent conjugates normalized to uptake in iron-depleted media of the respective compound. Grey bars represent control conditions with TAFC. Blocking with [Fe]TAFC reduces cellular uptake due to competition with the natural substrate of the MirB transporter. Fe (+) represents iron-replete conditions, which decreases biosynthesis of the MirB transporter. (**B**) Competition assay of [^68^Ga]Ga‐TAFC blocked with iron-containing fluorophore compounds in iron-depleted minimal medium. Values that are lower compared to the [^68^Ga]Ga‐TAFC uptake of control (grey bars), indicate a specific interaction of the fluorophore conjugate with the MirB transporter. Data of [Fe]DAFC-Cy5 are adapted from reference^18^.

Uptake assays and competition studies in *A. fumigatus* hyphae are summarized in Fig. 2. Values are normalized to the uptake of each gallium-68 labelled siderophore-conjugate, respectively. Uptake of DAFC-fluorophore conjugates by MirB should have decreased during competition with [Fe]TAFC or in iron-sufficient media, which causes transcriptional repression of siderophore uptake^22^. This was the case for [^68^Ga]Ga-DAFC-NBD and -Ocean Blue indicating MirB-dependent uptake. However, this was not the case for [^68^Ga]Ga-DAFC-BODIPY, -SiR, -Cy5 and -TAMRA indicating lack of uptake or unspecific binding to the hyphal surface. Moreover, [^68^Ga]Ga-DAFC-FITC showed only minor reduction of cellular accumulation in these blocking experiments.

In the ferrous form, all fluorescent conjugates were able to inhibit uptake of [^68^Ga]Ga-TAFC in competition experiments, confirming interaction with the MirB transporter. Thereby most compounds showed a comparable blocking to [Fe]TAFC except for [Fe]DAFC-NBD (more efficient blocking) and [Fe]DAFC-Bodipy (less efficient blocking).

#### Utilization of siderophore-conjugates by *ΔsidA/ftrAA. fumigatus*

**Figure 3:**
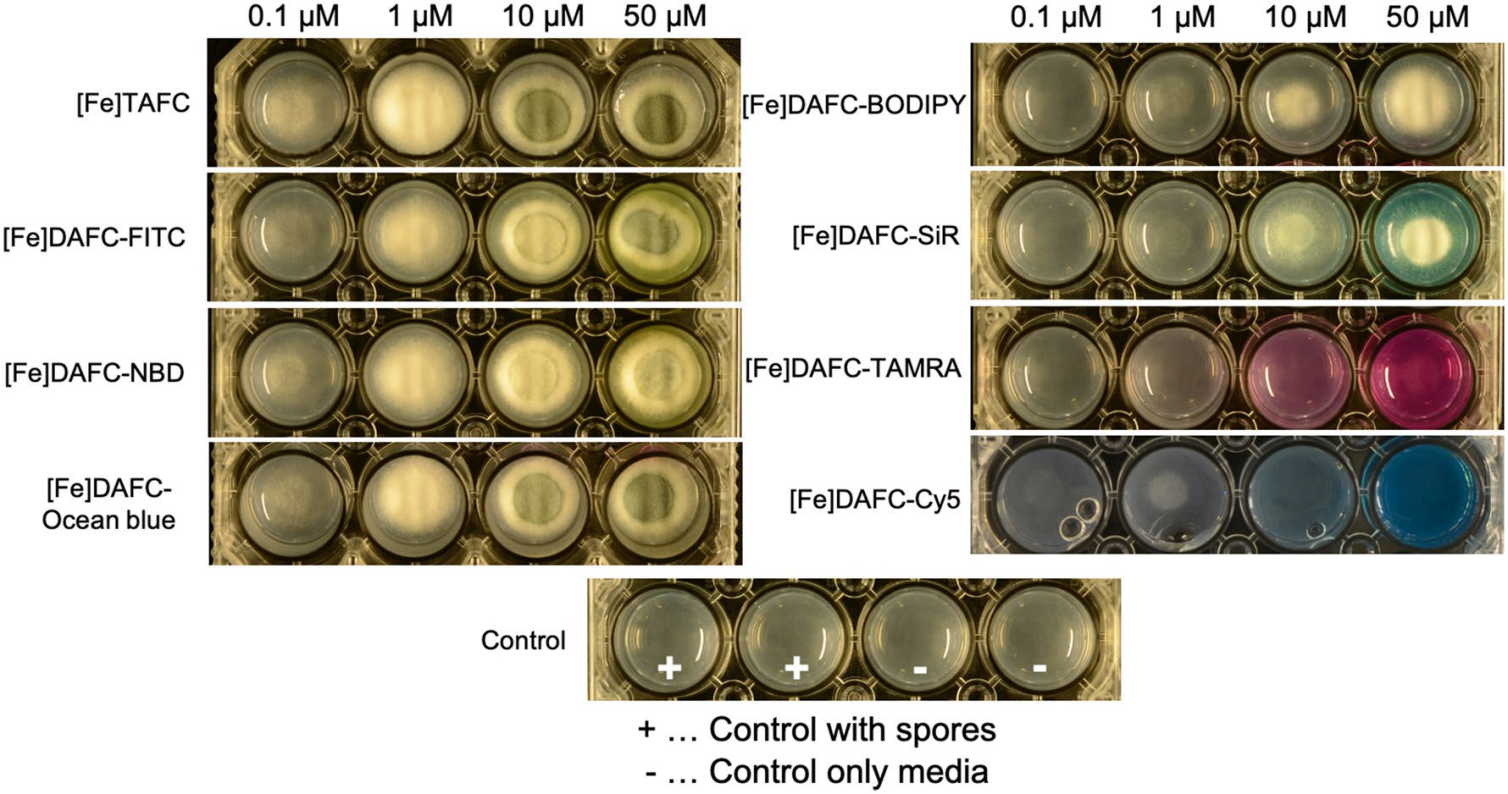
Growth of *A. fumigatus* mutant strain *ΔsidA/ΔftrA* after 48 h incubation at 37 °C on iron‐depleted Aspergillus minimal medium containing different iron-fluorophore conjugates at increasing concentrations (from right to left 0.1; 1; 10; 50 µM). Whitish mycelia result from hyphal growth, while green colouring reflects the formation of conidia. The bottom row shows growth without siderophores demonstrating that this mutant strain is not able to grow without usable siderophores. Data of [Fe]DAFC-Cy5 from reference^18^.

Utilization experiments already revealed growth induction at 0.1 µM for [Fe]TAFC (control) and sporulation at 10 µM (Fig. 3).[Fe]DAFC-FITC, -Ocean Blue and -NBD resulted in similar growth rates with corresponding sporulation. [Fe]DAFC-BODIPY and -SiR supported growth at 1 µM but did not induce sporulation, even when availability was raised to 50 µM.[Fe]DAFC-Cy5 promoted some growth at 1 µM but led to complete growth arrest above 50 μM, suggesting a concentration dependent inhibitory effect^18^. Interestingly, [Fe]DAFC-TAMRA did not support appreciable growth even at the highest possible concentration. Taken together these data indicate that all siderophore conjugates except [Fe]DAFC-TAMRA can be efficiently utilized as iron carriers by *A. fumigatus*.

#### Fluorescence microscopy

**Figure 4:**
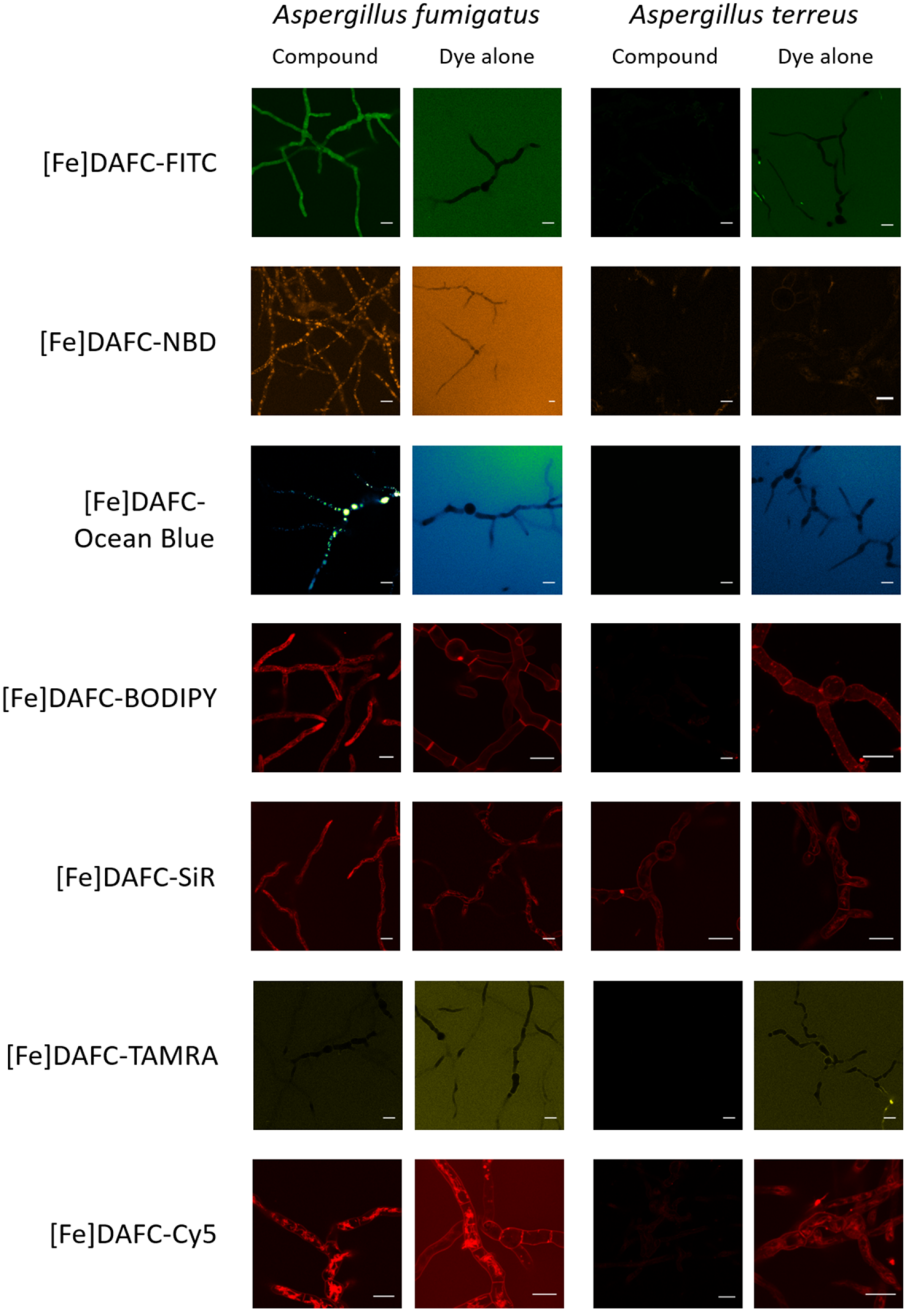
Internalisation and subcellular localisation pattern of fluorescent siderophore conjugates in *A. fumigatus* and *A. terreus*. [Fe]DAFC-FITC, -Ocean Blue, -NBD and –BODIPY are specifically internalised by *A. fumigatus*—but not by *A. terreus*—and accumulate in the cytoplasm, vacuoles and internal membranes, respectively. [Fe]DAFC-SiR and –Cy5 show pronounced internalisation into *A. fumigatus*, however, do leak into *A. terreus* by a MirB-independent mechanism. Interestingly, [Fe]DAFC-TAMRA is not internalised to detectable levels by either species. “Dye alone” controls—using identical molarities, incubation and imaging conditions—showed that only BODIPY, SiR and Cy5 are able to rapidly cross the cell wall matrix and plasma membrane barrier. Scale bars, 10 μm.

Live-cell imaging revealed heterogeneous results not only in terms of overall uptake of the [Fe]DAFC conjugates and dye-dependent uptake dynamics, but also in relation to the subcellular distribution pattern of the internalised molecules. Since [Fe]TAFC requires the MirB transporter to cross the plasma membrane, experiments with *A. terreus* which lacks MirB orthologs^17^, can be used as a control for potential unspecific, MirB-independent uptake. In addition, application of the free, non-conjugated fluorescent dye alone was used as control for siderophore-independent dye uptake (Fig. 4).

All fluorescent conjugates were efficiently internalised by *A. fumigatus* germlings and showed distinct subcellular localisation patterns, except [Fe]DAFC-TAMRA which remained outside under all tested conditions. [Fe]DAFC-BODIPY, -Cy5 and -SiR accumulated into longitudinal structures reminiscent of fungal mitochondria. [Fe]DAFC-NBD and -Ocean Blue localised into big circular structures, most likely vacuoles. [Fe]DAFC-FITC evenly distributed throughout the cytoplasm but also accumulated in circular structures. The control experiments with *A. terreus* confirmed that efficient uptake requires MirB because six of the seven siderophore conjugates were not internalised by germlings of this species. Only [Fe]DAFC-SiR produced weak intracellular signals suggesting unspecific uptake by an unknown passive mechanism. Application of fluorescent dyes alone showed that FITC, Ocean Blue, NBD and TAMRA did penetrate the cell wall matrix but did not enter the cell during the observation time. In contrast, unconjugated BODIPY, SiR and Cy5 internalised rapidly, most likely via endocytosis across the plasma membrane. There was no obvious distinction between *A. fumigatus* and *A. terreus*. Exemplary, NaN_3_ and [Fe]TAFC blocked cellular accumulation of [Fe]DAFC-Ocean Blue, indicating energy-dependant uptake by a membrane transporter, most likely MirB (Fig. S3).

**Figure 5:**
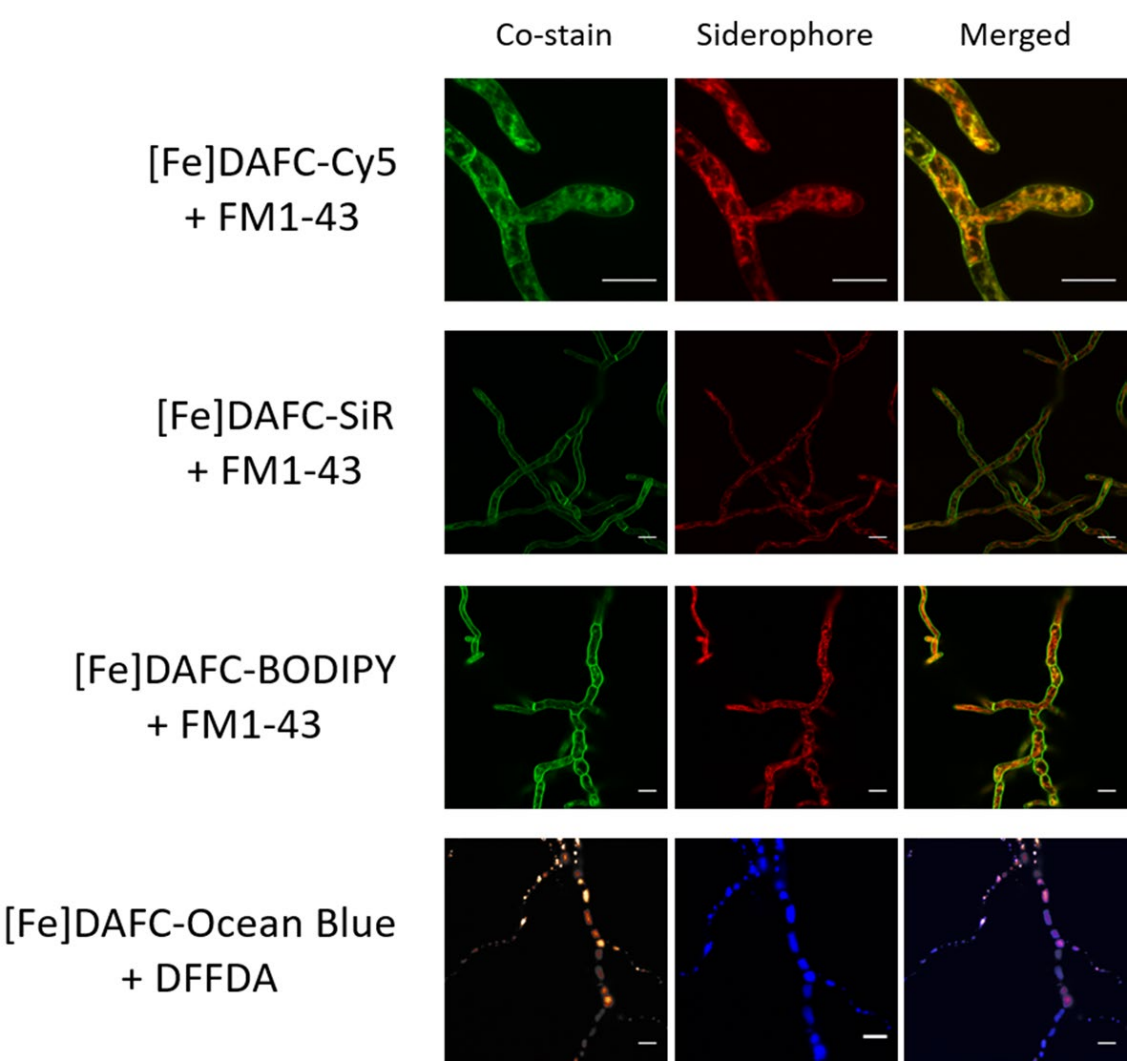
Co-staining with FM1-43 (green, mitochondria) and DFFDA (hot orange, vacuoles) confirmed the subcellular accumulation of different siderophore conjugates to fungal mitochondria and vacuoles, respectively. Scale bars, 10 µM. Supplementary Video S1 shows a time-lapse sequence of [Fe]DAFC-Cy5/FM1-43 co-labelling and supplementary Figure S2 shows Calcolfuor White/FM1-43 co-staining.

Co-staining with organelle-specific fluorescent dyes was used to confirm the localisation of internalised [Fe]DAFC conjugates. For instance, the lipophilic plasma membrane marker FM1-43, becomes endocytosed and distributes into mitochondrial and vacuolar membranes over time^3^. The mitochondria of filamentous fungi are longitudinal organelles that tend to accumulate near the tip of actively growing hyphae^5^ to support the high metabolic activity of the “Spitzenkörper”^3,23^. FM1-43 co-staining confirmed the suspected localisation of [Fe]DAFC-Cy5, -BODIPY and -SiR to mitochondria (Fig. 5). The fluorescent vacuolar marker DFFDA perfectly aligned with the subcellular localisation of [Fe]DAFC-Ocean Blue confirming its accumulation in vacuoles (Fig. 5).

## Discussion

Several attempts have been made to target the siderophore iron acquisition system with fluorescent probes. Desferoxamin B (DFO B) has been labelled with nitrobenzofuran (NBD) to acquire novel insight into the siderophore methabolism of the important plant pathogenic fungus *Ustilago maydis*^24^. The same DFO B-NBD conjugate has also been used to investigate *Plasmidium falciparum* infections. This modification allowed to overcome major drawbacks in the therapy of infected erythrocytes and also allowed to measure cellular uptake of the siderophore^25^. Doyle and colleagues modified FsC with NBD to perform fluorescence microscopy on A*. fumigatus* in order to visualize siderophore uptake into hypha^26^.

The current study focussed on the uptake properties of different fluorescent [Fe]DAFC conjugates that specifically target *A. fumigatus* due to its specific interaction with the MirB transporter.

Conjugation of the different fluorescent dyes resulted in high purity compounds with acceptable yields, depending on the conjugation strategy. As expected, they all showed a high dependency to size and charge of the fluorophore. Notably, conjugation increased water solubility of all compounds, compared to the dye alone, allowing a reduction of the amount of organic solvent used for live-cell imaging. Uptake assays also revealed different values depending on the dye. Based on previous findings we postulated that highly charged molecules have a tendency towards unspecific binding to the outer cell wall of the hyphae^17,18^. This phenomenon was indeed observed for [^68^Ga]Ga-DAFC-BODIPY, -TAMRA, -Cy5 and –SiR, which showed no decrease when blocked with [Fe]TAFC or in iron-sufficient media. As they all are charged molecules from + 1 to -1, interaction with the charged fungal cell wall matrix is likely. On the other hand, all conjugates showed a decrease of [^68^Ga]Ga-TAFC uptake in competition assays, indicating specific interaction with the MirB transporter. [^68^Ga]Ga-DAFC-FITC, -NBD and -Ocean Blue showed reasonable uptake which could be blocked and all resulted in a decrease of [^68^Ga]Ga-TAFC uptake in competition assays. These findings were further supported by utilization assays in which a comparable growth promotion was observed for all compounds except [Fe]DAFC-TAMRA.

Live-cell imaging visualized internalization of all siderophore conjugates by *A. fumigatus* with the exception of [Fe]DAFC-TAMRA. “Dye alone” controls revealed that Cy5, BODIPY and SiR are able to enter the cells passively, confirming on the other hand that the internalisation of [Fe]DAFC-FITC, -Ocean Blue and—NBD depends on active transport. The direct comparison to *A. terreus,* which lacks the MirB transporter^17^, illustrated the specificity of [Fe]DAFC conjugates for *A. fumigatus*. Except for [Fe]DAFC-SiR, where were a general non-specific uptake of the dye in both conjugated and unconjugated form was observed, all other fluorescent conjugates lacked detectable uptake into *A. terreus* germlings. Upon uptake in *A. fumigatus*, the different siderophore conjugates showed heterogeneous subcellular accumulation. [Fe]DAFC-BODIPY, -SiR and –Cy5 localises to the plasma membrane and predominantly to mitochondria as confirmed by FM1-43 co-staining^3,18^. [Fe]DAFC-NBD and -Ocean Blue exclusively accumulated in vacuoles as confirmed by DFFDA co-staining, whereas [Fe]DAFC-FITC remained in the cytoplasm^5,27,28^.

Taken together, the results show that the modification of TAFC with a variety of fluorescent dyes differentially affects cellular uptake and storage behaviour of the individual conjugates. The fact that different fluorescent moieties resulted in different subcellular localization of siderophore conjugates demands cautious interpretation of the cellular fate of siderophores after uptake via fluorescent labelling. Our studies underline that it is very important to consider that different fluorophores show different uptake behaviour and should be chosen wisely^29^, especially in terms of unspecific binding of the fluorophore itself. Importantly, due to the high specificity of the MirB transporter for TAFC, these dyes are also selective for *A. fumigatus*. Consequently, these fluorescent probes allow specific labelling of *A. fumigatus* in mixed cultures, e.g. containing different microbial or mammalian cells. Moreover, these probes can be used to visualization iron starvation in *A. fumigatus* as their uptake is repressed by iron. Furthermore, these probes allow identification of compounds that interact with the siderophore transporter via competition assays.

## Conclusion

Overall, this study demonstrates that fluorescent dyes can be coupled to the siderophore DAFC while retaining its recognition and uptake by the siderophore-transporter MirB in *A. fumigatus*. Furthermore, additional radiolabelling of the conjugates is possible under mild conditions, to facilitate in vitro and in vivo applications. Since TAFC is specific for the MirB transporter, the described compounds can be used for species-specific fluorescence labelling. Furthermore, by introducing various fluorescent dyes, different cellular compartments can be enlightened at different wavelengths. This structure-related information is also valuable for the development of antifungal siderophore conjugates that might be used for the treatment of *A. fumigatus* infections.

## Supplementary information

Supplementary Information

Supplementary Video S1
