# Supplementary material for: Live-cell imaging with Aspergillus fumigatus-specific fluorescent siderophore conjugates
Source: Sci Rep. 2020 Sep 23;10:15519. doi: 10.1038/s41598-020-72452-2 (PMC7511942; doi:10.1038/s41598-020-72452-2)
Supplement: Supplementary file 1 — Supplementary Information [file 41598_2020_72452_MOESM1_ESM.pdf]

SUPPLEMENTARY MATERIAL

Live-cell imaging with *Aspergillus fumigatus*-specific fluorescent siderophore conjugates

Joachim Pfister[1], Alexander Lichius[2], Dominik Summer[1], Hubertus Haas[3], Thines Kanagasundaram[4], Klaus Kopka[4], Clemens Decristoforo[*1]

MALDI-TOF MS: Matrix-assisted laser desorption/ionization time -of-flight mass spectrometry was performed on a Bruker microflex™ bench-top MALDI-TOF MS (Bruker Daltonics, Bremen, Germany). Samples were prepared on a micorscout target (MSP96 target ground steel BC, Bruker Daltonics) using dried-droplet method and α-cyano-4-hydroxycinnamic acid (HCCA, Sigma-Aldrich, Handels GmbH, Vienna, Austria) as matrix. All spectra were recorded by summarizing 600 laser shots per spot and Flex Analysis 2.4 software was used for data processing.

Analytical RP-HPLC: Reversed-phase (RP) high-perfomance Liquid chromatography (HPLC) analysis was carried out using the following instrumentation: UltiMate 3000 RS UHPLC pump, UltiMate 3000 autosampler, UltiMate 3000 Variable Wavelenght Detector; UV detection at λ = 220nm (Dionex, Germering, Germany) Radio-detector (Gabi Star, Raytest; Straubenhardt, Germany) using Jupiter 5 µM C_{18} 300 Å 150 x 4.6 mm (Phenomenex Ltd. Aschaffenburg, Germany) as column with acetonitrile (ACN)/H_2O/0.1% trifluoroacetic acid (TFA) as mobile phase; flow rate of 1 mL/min;

Gradient A: 0.0–3.0 min 10% ACN, 3.0–16.0 min 10–60 % ACN, 16.0–18.0 min 60% ACN, 18.0–18.1 min 60–10% ACN, 18.1–22.0 min 10% ACN.

Gradient B: 0.0–3.0 min 10 % ACN, 3.0–16.0 min 10–100 % ACN, 16.0–18.0 min. 100 % ACN, 18.0-18.1 min 100-10 % ACN, 18.1–22.0 min. 10 % ACN

Preparative RP-HPLC. Sample purification via RP-HPLC was carried out on a Gilson 322 Pump with a Gilson UV/VIS-155 detector (UV detection at λ = 220 nm) using a PrepFC™ automatic fraction collector (Gilson, Middleton, WI, USA), Eurosil Bioselect Vertex Plus 30 x 8 mm 5 µm C_{18}A 300Å pre-column and Eurosil Bioselect Vertex Plus 300 x 8 mm 5 µm C_{18}A 300Å column (Knauer, Berlin, Germany) and following ACN/H_2O/ 0.1 % TFA gradients with a flow rate of 2 mL/min:

Gradient 1: 0.0–1.0 min 0 % ACN, 1.0–35.0 min 0–50 % ACN, 35.0–36.0 min 50 % ACN, 36.0–36.1 min 50–0 % ACN, 36.1–43.0 min 0 % ACN

Gradient 2: 0.0–1.0 min 20 % ACN, 1.0–36.0 min 20–100 % ACN, 36.0–40.0 min 100 % ACN, 40.0–40.1 min. 100–20 % ACN, 40.1–47.0 min. 20 % ACN,

Gradient 3: 0.0–1.0 min 10% ACN, 1.0–36.0 min 10–80 % ACN, 36.0–37.0 min 80 % ACN, 37.0–37.1 min 80-10% ACN; 37.1–43.0 min 10% ACN

Precursor preparation:

[Fe]Fusarinin C ([Fe]FsC) was obtained by fungal culture according to Schrettl *et al* [20]. *Aspergillus fumigatus* mutant strain *ΔSidG* (which lacks the enzyme for acetylation of FsC) was seeded ($1*10^6$ Spores/mL) in 200 mL iron depleted minimal medium, incubated for 28 h at 37°C and shaking at 200 rpm. After filtering of the culture supernatant, $FeSO_4$ was added to a final concentration of 10 mM to get a red coloured solution. The filtrate was subsequently loaded to a Reveleris silica flash cartridge (C18; 40 µm; 12 g; column volume (CV) of 18 mL; BÜCHI Labortechnik AG, Flawil, Switzerland) by using a REGLO tubing pump (Type ISM795, Ismatec SA, Glattbrugg-Zürich, Switzerland) with a flow rate of 10 mL/min. Fixed [Fe]FsC on the cartridge was washed with 2 CV of water and then eluted with 5 CV of methanol. After evaporation of the organic solvent approximately 70 mg of [Fe]FsC could be obtained as a red-brown coloured solid with a purity of >90% confirmed by analytical RP-HPLC (gradient A t_R = 9.10 min) and the product was used for synthesis without further purification. MALDI-TOF-MS: *m/z* [M+H] = 780.68 [$C_{33}H_{51}FeN_6O_{12}$; exact mass: 779.63 (calculated)]

Acetylation of [Fe]Fusarinin C:

To acetylate [Fe]FsC 30 mg (38 µmol) dissolved in 500 µL water was rocked with 20 µL (0.2 µmol) of acetic anhydride for 2 min at room temperature and intense shaking. Resulting products mono-, di-, and triacetylfusarinin C were immediately purified via preparative RP-HPLC using gradient 1 to collect N,N´-diacetylfusarinine C ([Fe]DAFC, t_R = 22.3 min) in high purity (> 95%) followed by lyophilization. MALDI-TOF-MS: *m/z* [M+H] = 864.01 [$C_{37}H_{55}FeN_6O_{14}$; exact mass: 863.70 (calculated)] [12]

Conjugation of the fluorophores:

Conjugation of FITC.

[Fe]DAFC (10.0 mg, 11.6 µmol) was dissolved in dry DMF and after adding 1.1 equivalent (4.9 mg, 12.7 µmol) of Fluorescein 5-isothiocyanate (Sigma Aldrich, Vienna, Austria), pH 9 was adjusted using DIPEA and the reaction was stirred for one hour at ambient temperature. The reaction solution was directly purified by preparative RP-HPLC (gradient 2, t_R = 27.9 min) to give a yellow-brownish coloured solid after freeze drying. Analytical data: [Fe]DAFC-FITC 8.8 mg [7.02 µmol, 61%]; RP-HPLC gradient A; t_R = 12.4 min; MALDI TOF-MS: m/z [M+H] = 1253.96 [$C_{58}H_{66}FeN_7O_{19}S$; exact mass: 1253.09 (calculated)].

Conjugation of BODIPY 630/650

For the conjugation of BODIPY 630/650 carboxylic acid 0.88 mg (1.54 µmol, Lumiprobe GmbH, Hannover, Germany) was dissolved in 500 µL dry DMF and after addition of 1.1 equiv. (0.7 mg, 1.7 µM) HATU the mixture was left for 10 min at room temperature to activate the carboxylic acid. Subsequently 2 equiv. of [Fe]DAFC (2.66 mg, 3.08 µmol) were added and pH was adjusted to pH= 8-9 with DIPEA. The reaction was stirred for further 30 min and the end were confirmed by analytical RP-HPLC. Product was immediately purified via preparative RP-HPLC (Gradient 2 t_R = 26.7 min) and freez-dried to give a dark blue coloured solid. Analytical data: 1.3 mg [1.00 µmol; 65% yield] RP-HPLC gradient A t_R = 17.5 min, MALDI TOF-MS: *m/z* [M+H] = 1297.28 [$C_{60}H_{70}BF_2FeN_8O_{16}S$; exact mass: 1295.95 (calculated)]

Conjugation of hexanoyl-NBD

NBD (6-(7-Nitrobenzofurazan-4-ylamino)hexanoic acid) (10.22 mg; 34.73 µmol, Sigma Aldrich, Vienna, Austria) was dissolved in DMF and incubated with HATU (13.21 mg; 34.76 µmol) for 10 minutes to activate the carboxylic acid. [Fe]DAFC (10.0 mg; 11.58 µM) and 30 µL of DIPEA were added to the solution and stirred for 1 hour at room temperature. End of the reaction was confirmed by RP-HPLC Finally the product was purified by preparative RP-HPLC (gradient 2, t_R= 25.7 min) Analytical data: [Fe]DAFC-NBD 8.12 mg [7.12 µmol, 60 % yield]; RP-HPLC gradient B: t_R= 13.7, MALDI TOF-MS: m/z [M+H]+ = 1140.23 [$C_{49}H_{67}FeN_{10}O_{18}$; exact mass: 1139.95 (calculated)].

Conjugation of 5-TAMRA-SE

[Fe]DAFC (3.2 mg, 3.6 µmol) was dissolved in anhydrous DMF and 1.5 mg (2.8 µmol) 5-TAMRA-NHS ester (Bio-Techne Ltd, Abingdon, UK) dissolved in dry DMF was added and after pH adjustment (pH 8.5) with DIPEA, the reaction mixture was stirred at RT under light exclusion for three hours. After conjugation, confirmed by RP-HPLC the reaction solution was purified by preparative RP-HPLC (Gradient 3, t_R = 25.25 min) to give a pink coloured solid after lyophilisation. Analytical data: [Fe]DAFC-TAMRA 0.72 mg [0.56 µmol, 20 % yield]; RP-HPLC gradient B, t_R = 11.45 min; MALDI-TOF-MS: m/z [M+H] = 1276.19 [$C_{62}H_{76}FeN_8O_{18}$; exact mass: 1277,49 (calculated)].

Conjugation of Ocean Blue SE

[Fe]DAFC (4.9 mg, 5.7 µmol) was dissolved in anhydrous DMF and 1.5 mg (4.4 µmol) Ocean blue-NHS ester (Bio-Techne Ltd, Abingdon, UK) was added and after pH adjustment (pH 8.5) with DIPEA, the reaction mixture was stirred at RT under light exclusion for three hours. Conjugation was confirmed by RP-HPLC and the reaction solution was purified by preparative RP-HPLC (Gradient 3, t_R = 22.36 min) to give a light brown coloured solid after lyophilisation. Analytical data: [Fe]DAFC-Ocean blue 0.89 mg [0.81 µmol, 18 % yield]; RP-HPLC gradient B, t_R = 11.68 min; MALDI-TOF-MS: m/z [M+H] = 1088.30 [$C_{47}H_{57}F_2FeN_6O_{18}$; exact mass: 1087.31 (calculated)].

Conjugation of Rose Bengal

For the conjugation of RoseBengal 11.8 mg (12.1 µmol; Sigma Aldrich, Vienna, Austria) was dissolved in 500 µL dry DMF and after addition of 1.1 equiv. (5.1 mg, 13.3 µM) HATU and 10µL DIPEA, the mixture was left for 10 min at room temperature to activate the carboxylic acid. Subsequently 5.0 mg of [Fe]DAFC (5.7 µmol) were added and the reaction was stirred for further 30 min. The end of the reaction was confirmed by analytical RP-HPLC. Hereafter the product was isolated via preparative RP-HPLC (Gradient 2: t_R= 25.8 min) and freez-dried as a red powder. Analytical data: [Fe]DAFC-RoseBengal 2.0 mg [1.09 µmol; 20% yield]; RP-HPLC gradient B t_R = 16.6 min MALDI TOF-MS: *m/z* [M+H] = 1819.71 [$C_{57}H_{57}FeI_4N_6O_{18}$; exact mass: 1819.8 (calculated)]

Conjugation of SiR

[Fe]DAFC succ (2.55 mg, 2.6 µmol) was dissolved in anhydrous DMF and 1.1 mg (2.6 µmol) 10-(5-amino-2-methylphenyl) silicon-rhodamine (prepared in house [19]) dissolved in dry DMF was added and after pH adjustment (pH 8.5) with DIPEA, the reaction mixture was stirred at RT

under light exclusion for one hour. After conjugation, confirmed by RP-HPLC the reaction solution was purified by preparative RP-HPLC (Gradient 3 t_R = 25.21 min) to give a dark blue coloured solid after lyophilisation. Analytical data: [Fe]DAFC-succ-SiRo 0.80 mg [0.36 µmol, 22 % yield, purity >95%]; RP-HPLC gradient B, t_R = 12.46 min; MALDI-TOF-MS: m/z [M+H] = 1359.54 [$C_{67}H_{89}FeN_9O_{16}Si$; exact mass: 1360.40 (calculated)].

Demetallation

For the purpose of iron removal, the corresponding conjugates were dissolved in 1 mL H_2O/organic solvent (e.g. EtOH) 10-70 % (v/v) and 1 mL of aqueous Na_2EDTA solution (200 mM) was added. The resulting mixtures were stirred under light exclusion for 4 h at ambient temperature followed by preparative RP-HPLC purification to give the iron free fluorescent conjugates after lyophilisation.

DAFC-FITC: 0.5 mg [yellow, 0.42 µmol, purity >98%], gradient 1 (t_R = 28.5 min); Analytical data: RP-HPLC gradient A; t_R = 12.6 min; MALDI TOF-MS: m/z [M+H] = 1201.06 [$C_{58}H_{69}N_7O_{19}S$; exact mass: 1200.27 (calculated)].

DAFC-BODIPY 630/650: 0.4 mg [blue, 0.3 µmol, purity >98%] gradient 2 (t_R = 26.4 min); Analytical data: RP-HPLC gradient B, t_R = 17.7min; MALDI TOF-MS: m/z [M+Na] = 1266.32 [$C_{60}H_{73}BF_2N_8O_{16}S$; exact mass: 1243.13 (calculated)]

DAFC-NBD: 4.1 mg [orange, 3.7 µmol; purity >90%] gradient 2 (t_R = 26.2 min); Analytical data: RP-HPLC gradient B, t_R = 14.2 min; MALDI TOF-MS: *m/z* [M+H] = 1089.09 [C $C_{49}H_{70}FeN_{10}O_{18}$; exact mass: 1087.13 (calculated)]

DAFC-TAMRA: 0.58 mg [pink, 0.47 µmol, purity >90%] gradient 3 (t_R = 24.9 min); Analytical data: RP-HPLC gradient B, t_R = 11.6 min; MALDI-TOF-MS: m/z [M+H] = 1223.44 [$C_{62}H_{79}N_8O_1$; exact mass: 1223.56 (calculated)]

DAFC Ocean Blue: 0.46 mg [light yellow, 0.43 µmol, purity >95%] gradient 3 (t_R = 23.1 min); Analytical data: RP-HPLC gradient B, t_R = 11.8 min; MALDI-TOF-MS: m/z [M+H] = 1025.4 [$C_{47}H_{60}F_2N_6O_{18}$; exact mass: 1024.29 (calculated)]

DAFC-Rose Bengal: 0.9 mg [rose, 0.5 µmol, purity >94%] gradient 2 (t_R = 25.3min); Analytical data: RP-HPLC gradient B, t_R = 16.6 min; MALDI TOF-MS: m/z [M+Na] = 1788.46 [$C_{60}H_{57}I_4N_6O_{18}$; exact mass: 1766.36 (calculated)]

DAFC-SiR: 0.27 mg [0.2 µmol, purity >80%]; Analytical data: TP-HPLC gradient B, t_R = 12.73 min ESI-Orbitrap-MS: m/z [M+H] = 1306.68 [$C_{67}H_{92}N_9O_{16}Si$; exact mass: 1306.66 (calculated)].

Radio-HPLC Chromatograms of ^{68}Ga-labelled siderophores

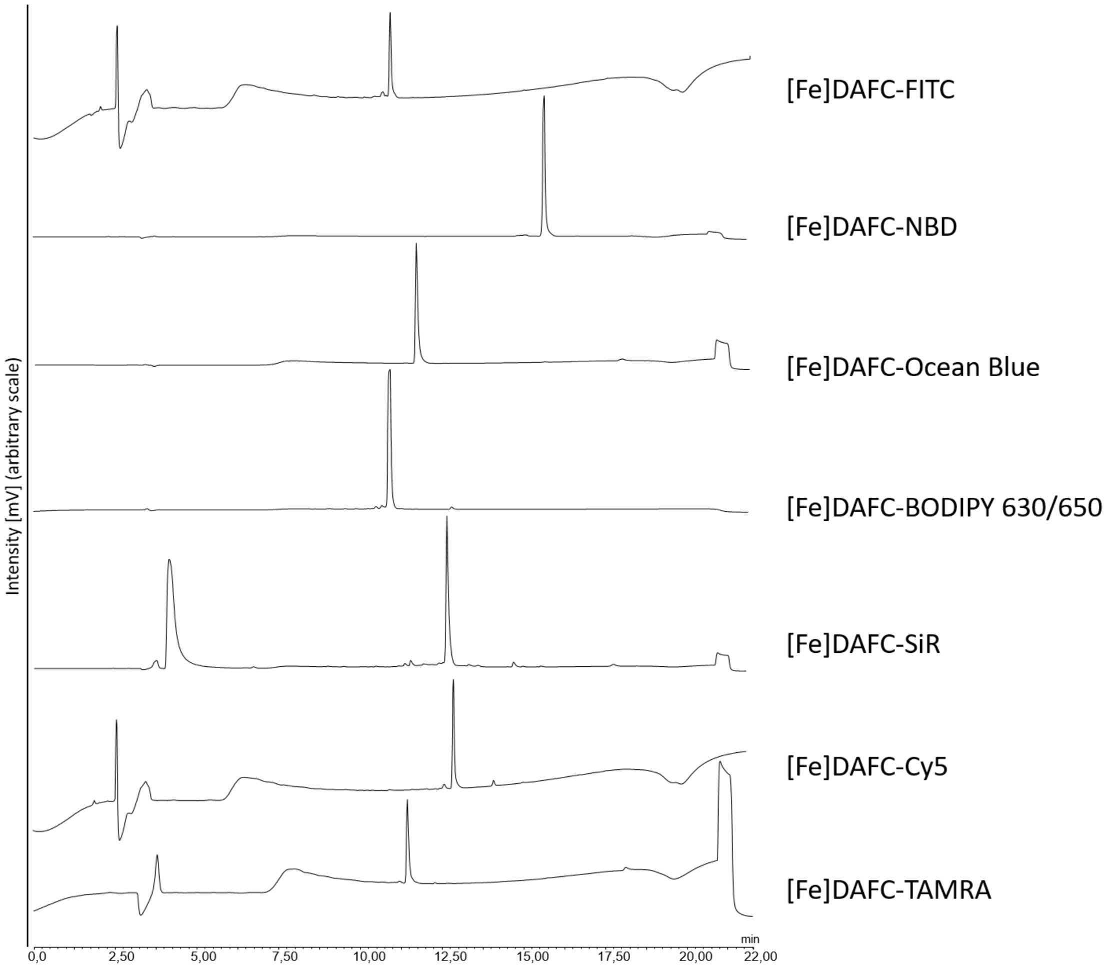

Figure S1: HPLC chromatograms of iron containing, fluorescent labelled siderophores; peaks with Rt<4 min origin of void volume / solvent

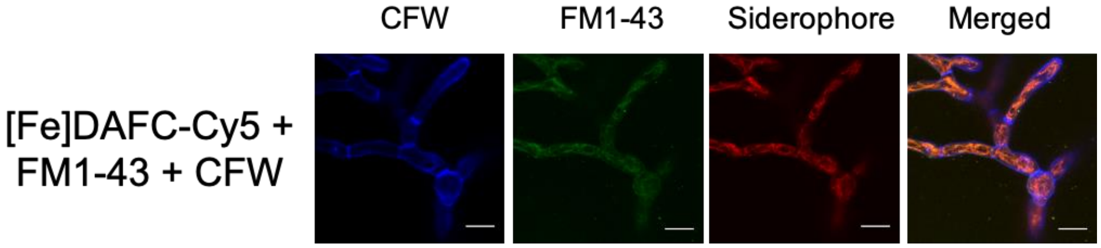

Figure S2: [Fe]DAFC-Cy5 co-stained with FM1-43 (green) and CFW (blue) and a merged image. Scale bar 10 µm

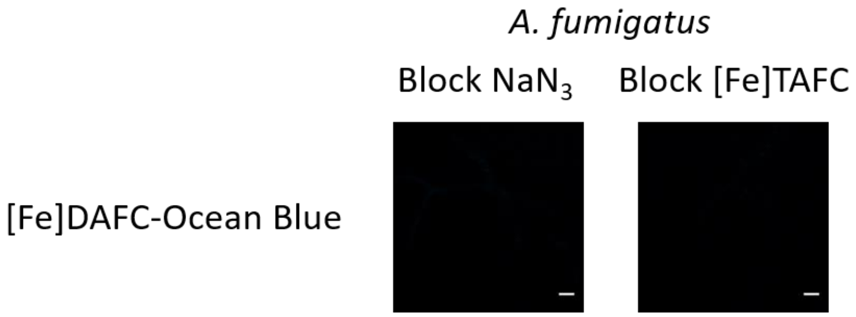

Figure S3: Live-cell image of [Fe]DAFC-Ocean Blue in *A. fumigatus*, pre-incubated with NaN_3 (inhibition of energy dependent mechanism) and [Fe]TAFC (saturation of the MirB transporter). Scale bar 10 µm.

Table S1. Live-cell imaging acquisition settings for the used fluorescent siderophore conjugates.

Fluorescent dye	Concentration [µM]	Excitation [nm]	Detection [nm]	Colour code
Calcofluor White	10	405	410-470	Blue
Ocean Blue	10	405	455-505	Blue/Green
FITC	10	488	500-550	Green
NBD	10	488	500-550	Yellow
DFFDA	10	496	505-555	Yellow
FM1-43	10	514	520-600	Green
TAMRA	10	562	570-620	Yellow
BODIPY	5	633	650-750	Red
SiR	10	633	655-705	Red
Cy5	2.5	633	640-690	Red
